# Concreteness and abstraction in everyday explanation

**DOI:** 10.3758/s13423-017-1299-3

**Published:** 2017-05-11

**Authors:** Christos Bechlivanidis, David A. Lagnado, Jeffrey C. Zemla, Steven Sloman

**Affiliations:** 1 Department of Experimental Psychology, University College London, 26 Bedford Way, London, WC1H 0AP UK; 2 Department of Cognitive, Linguistic & Psychological Sciences, Brown University, Providence, RI USA

**Keywords:** Explanation, Causal reasoning

## Abstract

A number of philosophers argue for the value of abstraction in explanation. According to these prescriptive theories, an explanation becomes superior when it leaves out details that make no difference to the occurrence of the event one is trying to explain (the explanandum). Abstract explanations are not frugal placeholders for improved, detailed future explanations but are more valuable than their concrete counterparts because they highlight the factors that do the causal work, the factors in the absence of which the explanandum would not occur. We present several experiments that test whether people follow this prescription (i.e., whether people prefer explanations with abstract difference makers over explanations with concrete details and explanations that omit descriptively accurate but causally irrelevant information). Contrary to the prescription, we found a preference for concreteness and detail. Participants rated explanations with concrete details higher than their abstract counterparts and in many cases they did not penalize the presence of causally irrelevant details. Nevertheless, causality still constrained participants’ preferences: They downgraded concrete explanations that did not communicate the critical causal properties.

What is the difference between a good description and a good explanation of an event? The former is an enumeration of the state of affairs at the time and place of the event while the latter is an account of why or how that event came to be. Consider a description of a car accident resulting in a pedestrian being injured. One can describe the location of the accident, the speed of the car, the conditions of the tires and the road. One could also refer to the make and color of the car, whether the car radio was on and even the clothes the driver was wearing, the color of the victim’s eyes, the number of nearby cars and pedestrians—an exhaustive array of facts that were true when the event in question took place. Arguably, the amount of information contained in a good description is constrained only by pragmatic considerations: the available time and space modulated by the needs and patience of the audience.

On the other hand, given that the central aim of explanation is to provide understanding—be it for purposes of diagnosis, prediction, or pure aesthetic pleasure (Keil, 2006)—only information in service of that aim should be included. Arguably, the color of the driver’s eyes in the above example does not enhance anyone’s understanding regarding the car accident, therefore it might be descriptively relevant but it is probably not explanatorily relevant.

Determining what is explanatorily relevant depends on the account of explanation one adopts, and philosophers have long debated the nature of explanation, especially in scientific practice (Hempel, 1965; Kitcher, 1981; Salmon, 1984; Strevens, 2008). A parallel question is whether explanation quality increases as accuracy increases. Unlike a description, whose quality is often contingent on the precision of its representation, there are reasons to believe that “abstracting” an explanation (i.e., removing certain details or decreasing its precision) might in fact improve it.

For some philosophers, abstraction signifies an undesirable departure from reality. On such views, an ideal explanation would mention every relevant factor at the highest degree of precision, but this quickly becomes unattainable either due to incomplete knowledge or to practical limitations. Cartwright (1983) argues that through abstraction, scientific explanations become false since they apply only in ideal conditions not found in nature. Railton (1981) argues that abstraction is a fair compromise, but a compromise nevertheless. For Nowak (1992), the distance from reality is progressively minimized by successive scientific theories: Starting from an abstract but false theory of a phenomenon, progress is achieved by adding more and more influencing factors and specifying them with more and more accuracy, such that the theory is brought closer and closer to reality.

Others, however, attribute value to abstraction. Jorland (1994), for example, thinks that abstraction improves explanations by leaving out nonessential factors, thus enabling “one to grasp the essence” (p. 274). Garfinkel (1981) explains that hyperconcrete explanations (i.e., explanations that contain too much detail) are overly sensitive to slight perturbations. Explaining an accident by referring to the car’s high speed, for example, is more robust than referring to the speed *and* the color of the driver’s shirt: while the former will explain multiple accidents, the latter will lead to different explanations for accidents that occurred at the same speed but in which the driver wore a blue, red, or purple shirt. Explanations that are too concrete are not merely “too good to be true” (i.e., impractical) but rather “too true to be good” (Garfinkel, 1981, p. 58).

Similarly, proponents of causal, and especially counterfactual, theories of explanation (Hitchcock & Woodward, 2003; Kuorikoski & Ylikoski, 2010; Strevens, 2007; Weslake, 2010) believe that an explanation can be improved even by leaving out terms that assert causal influence on the event to be explained (the explanandum). Specifically, Strevens (2007) argues that properly abstracted explanations are explanatorily superior to their concrete and, by definition, more accurate counterparts by focusing on difference makers (facts or events in the absence of which the explanandum would not occur). Thus, the criterion for mentioning a detail when explaining some phenomenon is whether that detail makes a difference to the phenomenon’s occurrence. When explaining why it took approximately 2 seconds for the apple falling from a tree to reach the ground, even if the gravitational pull from the moon did exert some influence on the apple, it did not change the fact that the flight of the apple lasted for approximately the time it did. On that basis, the removal of lunar influence from the terms mentioned in the explanation improves its quality. In short, Strevens (2007) distinguishes causal influence from difference making and argues that it is difference making that is key to explanatory quality. In a similar vein, Garfinkel (1981) points out that explanations are often intended to help the prevention of future occurrences of the explanandum. Details that make no difference have no use and may in fact hinder such practical goals.

Philosophical discussions often focus on explanations as used in science in an attempt to arrive at normative principles regarding abstraction. What principles do people use when evaluating everyday explanations? Is abstraction still valued? Do people choose to mention the exact speed of the car, or is it preferable to say that the car was moving fast or that the speed of the car fell within a certain range of values, all of which would still lead to the accident? Similarly, does including causally irrelevant but accurate information, such as the make of the car or even the color of the driver’s shirt, reduce the quality of the explanation?

Experimentally, Weisberg, Keil, Goodstein, Rawson, and Gray (2008) have shown that when judging behavioral explanations, people value the presence of neuroscientific details, even when these do not do any explanatory work (see also Fernandez-Duque, Evans, Christian, & Hodges, 2015). Their hypothesis is that psychological accounts that appeal to neuroscience—a lower level discipline dealing with phenomena that are more microscopic than psychology’s—generate an unwarranted sense of understanding (Trout, 2008; see also Hopkins, Weisberg, & Taylor, 2016). Another option, however, is that people do not have a bias toward neuroscience-based or even reductionist explanations per se but have a preference for concretization, for the detail and precision that a lower level explanation provides. In that case, one would expect to observe a preference for concreteness even when competing explanations do not differ in their level of reduction.

In the present set of experiments, we address these questions by obtaining ratings of competing explanations of everyday events that differ in the amount of information removed or abstracted from descriptions. At the extreme is what we call “irrelevant” explanations: explanations that contain all the information present in the description including causally irrelevant detail (i.e., information about events that do not plausibly exert any causal influence on the explanandum).^1^ The removal of irrelevant information yields what we call “concrete” explanations, containing only causally relevant information specified with a high degree of accuracy. Finally, by replacing precise descriptions with qualitative statements, we have constructed “abstract” explanations that specify only the difference makers, the events without which the explanandum would not occur.

## Experiments 1a and 1b

**Fig. 1:**
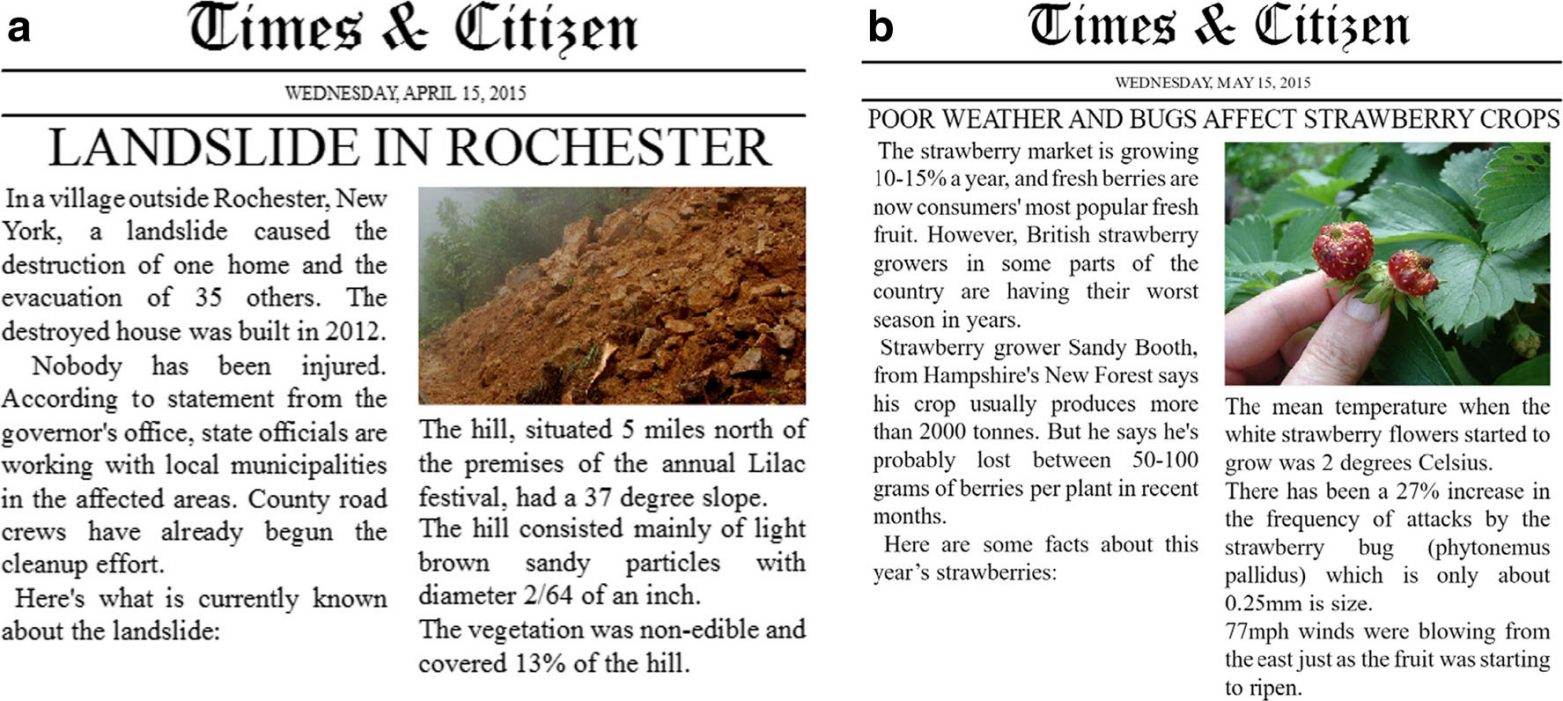
Stimuli used in Experiments 1a (*left*) and 1b (*right*). The information contained in the *right column* of each picture was shown again when participants were asked to rate the explanations

Participants were presented with a description of either a landslide (Experiment 1a) or a bad strawberry yield (Experiment 1b). To increase the believability of the descriptions, the stories were presented as newspaper reports (see Fig. 1). In addition, although the stories were made up, the information we used was copied from various specialized sources (e.g., geological surveys, pest management reports). After reading the report, participants were asked to rate the quality of three explanations that differed in their degree of concreteness (irrelevant, concrete, and abstract).

Following the philosophical views on the importance of causality and counterfactual dependence in explanation (Garfinkel, 1981; Hitchcock & Woodward, 2003; Strevens, 2007), we expected noncausally related details to be penalized by participants. Furthermore, given the level at which the question was posed (“Why was there a landslide?” rather than “Why did the landslide happen in this particular way?”), we expected participants to penalize details that had a causal influence but did not make a difference as to whether or not the landslide occurred, such as the exact diameter of soil particles. In contrast, if the tendency toward reductionism (Trout, 2008; Weisberg et al., 2008) is actually a particular case of a more general tendency for accuracy, then people should prefer the more detailed explanations.^2^

### Participants and materials

We recruited 61 participants for each of Experiments 1a and 1b through Amazon’s Mechanical Turk. The mean age was 32.9 years (*SD* = 11.6) in Experiment 1a and 31.7 years (*SD* = 11.1) in Experiment 1b. There were 35 females (57.4%) in Experiment 1a and 34 (55.7%) in Experiment 1b. In this and all experiments, participants were paid $0.50 for taking part, and the experiments were programmed in Adobe Flex 4.6 and conducted over the Internet (all experiments and the collected raw data can be seen at http://goo.gl/0rMOQd).

### Design and procedure

All participants rated all three explanations. After two introductory screens that welcomed participants and asked them to minimize disturbances during the experiment, participants saw the description of a landslide (Experiment 1a) or a strawberry harvest (Experiment 1b) presented as a newspaper report (see Fig. 1).

The landslide report included some noncritical information aimed at increasing the believability of the report (e.g., “Road crews have begun the cleanup effort”). The main causal factors were the slope, the consistency, and the vegetation of the hill. Three additional causally irrelevant facts described the color of the hill’s particles, the edibility of the vegetation, and the position of the hill relative to the premises of a local festival.^3^

Similarly, the strawberry report initially mentioned the strawberry market growth and went on to describe a bad strawberry season by referring to the temperature, the attacks of a strawberry bug, and the winds during ripening. The three causally irrelevant details with respect to the poor strawberry harvest were the size of the bug, the direction of the winds, and the color of the strawberry flowers.

In the next screen, participants were asked, based on the information contained in the newspaper report, to rate the quality of the three explanations by placing a marker on sliders that ranged from *poor* to *excellent*. The way explanations should be rated was left intentionally vague as we wanted participants to apply their own intuitive criteria. To make sure that participants were not treating the question as a memory test, the critical information from the newspaper report was repeated in this screen. The order in which explanations appeared on the screen was randomized for each participant.

The three explanations (see Table 1) differed in their degree of concreteness: They either repeated, omitted, or altered the information contained in the original newspaper report. In each scenario, six facts were manipulated. Three facts were included unchanged in the concrete and the irrelevant explanations but were abstracted in the abstract explanation. Another three were included only in the irrelevant explanation and were omitted from both the concrete and the abstract explanations. Thus, there were two critical comparisons: one between the concrete and the abstract explanations, with the latter containing the same information at a higher level of abstraction, and a second comparison between concrete and irrelevant explanations that differed only in the three additional but causally irrelevant facts contained in the latter.

**Table 1:** The three types of explanations used in Experiments 1a and 1b. Highlighted in bold here but not in the actual experiment are the differences between the explanations

	Experiment 1a	Experiment 1b
Abstract	The fact that the hill consisted mainly of **fine** sandy particles meant that the soil was unstable. The **sparse** vegetation did not withhold the rainwater causing soil erosion. Finally, the force of gravity acting down the **steep** slope overcame the resistance of friction, thus triggering the landslide.	The fact that the mean temperature when the strawberry flowers started to grow was **very low** caused some of the plants to freeze. The **significant** increase in attacks by the strawberry bug, which lays eggs on the leaves, stopped the development of many plants. Finally, the **extremely strong** winds uprooted many plants off their shallow roots.
Concrete	The fact that the hill consisted mainly of sandy particles with **diameters 2/64 of an** inch meant that the soil was unstable. The vegetation covering **13% of the hill** did not withhold the rainwater, causing soil erosion. Finally, the force of gravity acting down the **37 degree slope** overcame the resistance of friction, thus triggering the landslide.	The fact that the mean temperature when the strawberry flowers started to grow was **2 degrees Celsius** caused some of the plants to freeze. The **27%** increase in attacks by the strawberry bug, which lays eggs on the leaves, stopped the development of many plants. Finally, the **77-mph winds** uprooted many plants off their shallow roots.
Irrelevant	The fact that the hill, which **was 5 miles north of the premises of the annual Lilac festival**, consisted mainly of **light brown** sandy particles with diameters 2/64 of an inch meant that the soil was unstable. The **nonedible** vegetation covering 13% of the hill did not withhold the rainwater, causing soil erosion. Finally, the force of gravity acting down the 37 degree slope overcame the resistance of friction, thus triggering the landslide.	The fact that the mean temperature when the **white** strawberry flowers started to grow was 2 degrees Celsius caused some of the plants to freeze. The 27% increase in attacks by the **0.25-mm** strawberry bug, which lays eggs on the leaves, stopped the development of many plants. Finally, the 77-mph winds **blowing from the east** uprooted many plants off their shallow roots.

After rating the three explanations, participants were asked to report the causal relevance of each of the terms used in the explanations. For each of the nine terms (three concrete, three abstract, three irrelevant), participants were asked two questions intended to test whether causal relevance guides the evaluation of explanations: Did they agree with the assertion that the term “was a cause of the landslide/poor strawberry production” (henceforth: causal ratings), and did they agree with the assertion that the term “affected the particular way in which the landslide happened”/“affected particular aspects of this year’s poor strawberry production” (henceforth: causal influence ratings)? These questions also served as a validity check: They allowed us to verify whether our designation of certain terms as causally irrelevant agreed with people’s intuitions. The two versions of the causal question (causality and causal influence) were meant to capture potential differences between “what” and “how” causation (Gerstenberg, Goodman, Lagnado, & Tenenbaum, 2015). In other words, it may be the case that participants prefer concrete terms (e.g., 37 degrees slope) rather than abstract terms (e.g., steep slope), even though both adequately explain *what* happened (e.g., a landslide), because only the concrete term explains exactly *how it happened*, despite the fact this is not what is being asked.

### Results

**Fig. 2:**
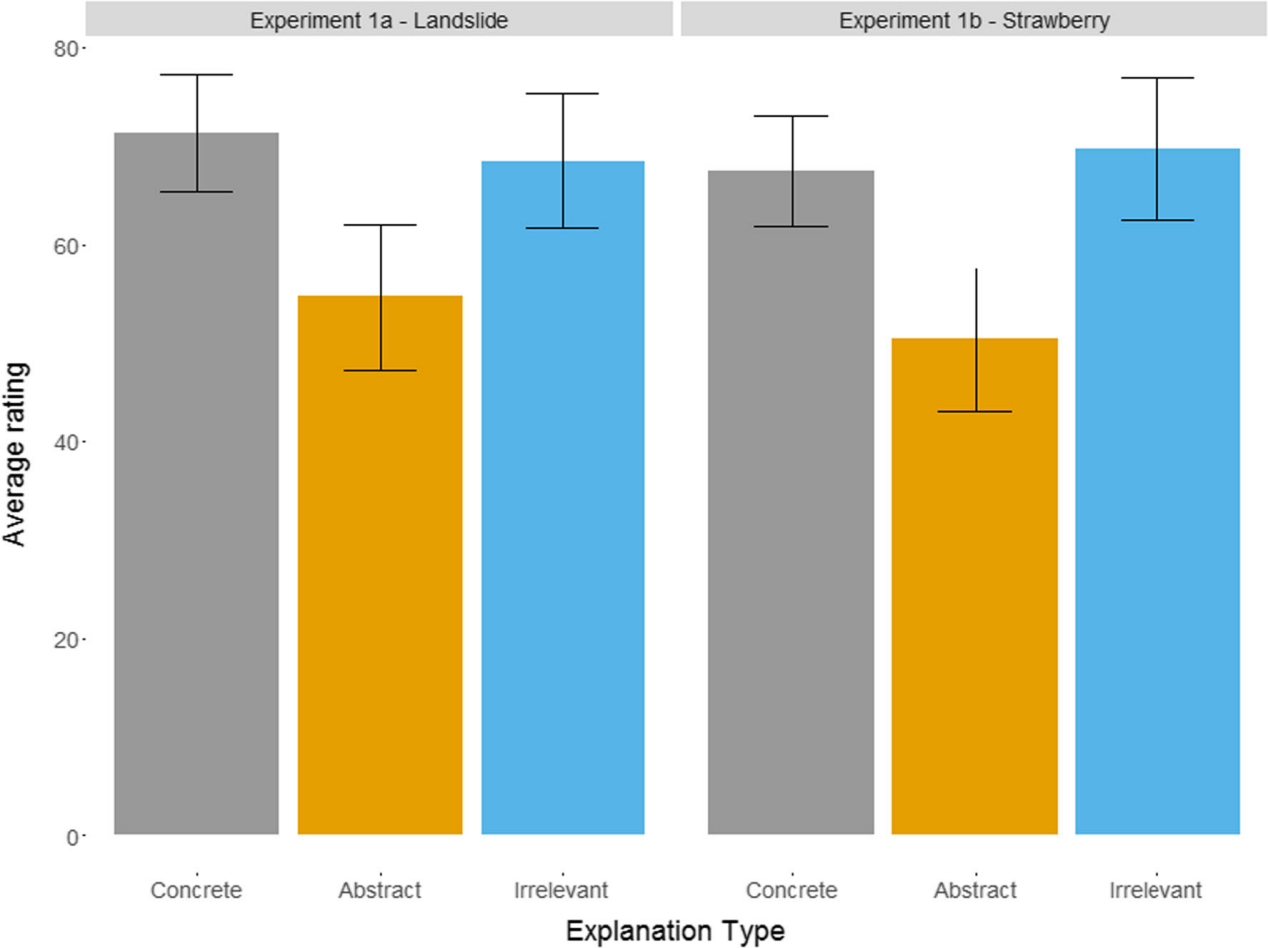
Mean explanation ratings for the three types of explanations in Experiments 1a and 1b averaged over participants (*error bars* represent 95% CI)

As shown in Fig. 2, the abstract explanations received the lowest ratings, while concrete and irrelevant explanations received roughly equal ratings. The pattern of results is identical in both experiments with a significant effect of explanation type: *F*(1.79, 107.5) = 6.23, *p* = .004, η^2^ = 0.07, for Experiment 1a, and *F*(1.65, 99.1) = 8.35, *p* = .001, η^2^ = 0.1, for Experiment 1b.

Follow-up paired *t* tests (Bonferroni adjusted) show no difference between concrete and irrelevant explanations for either experiment but a highly significant difference between concrete and abstract in both Experiment 1a, *t*(60) = 3.69, **p** < .001, *d* = 0.47, and Experiment 1b, *t*(60) = 4.12, *p* < .001, *d* = 0.53. Finally, irrelevant explanations were rated significantly higher than abstract in both Experiment 1a, *t*(60) = 2.36, *p* = .02, *d* = 0.3, and Experiment 1b, *t*(60) = 3.12, *p* = .003, *d* = 0.4.

**Fig. 3:**
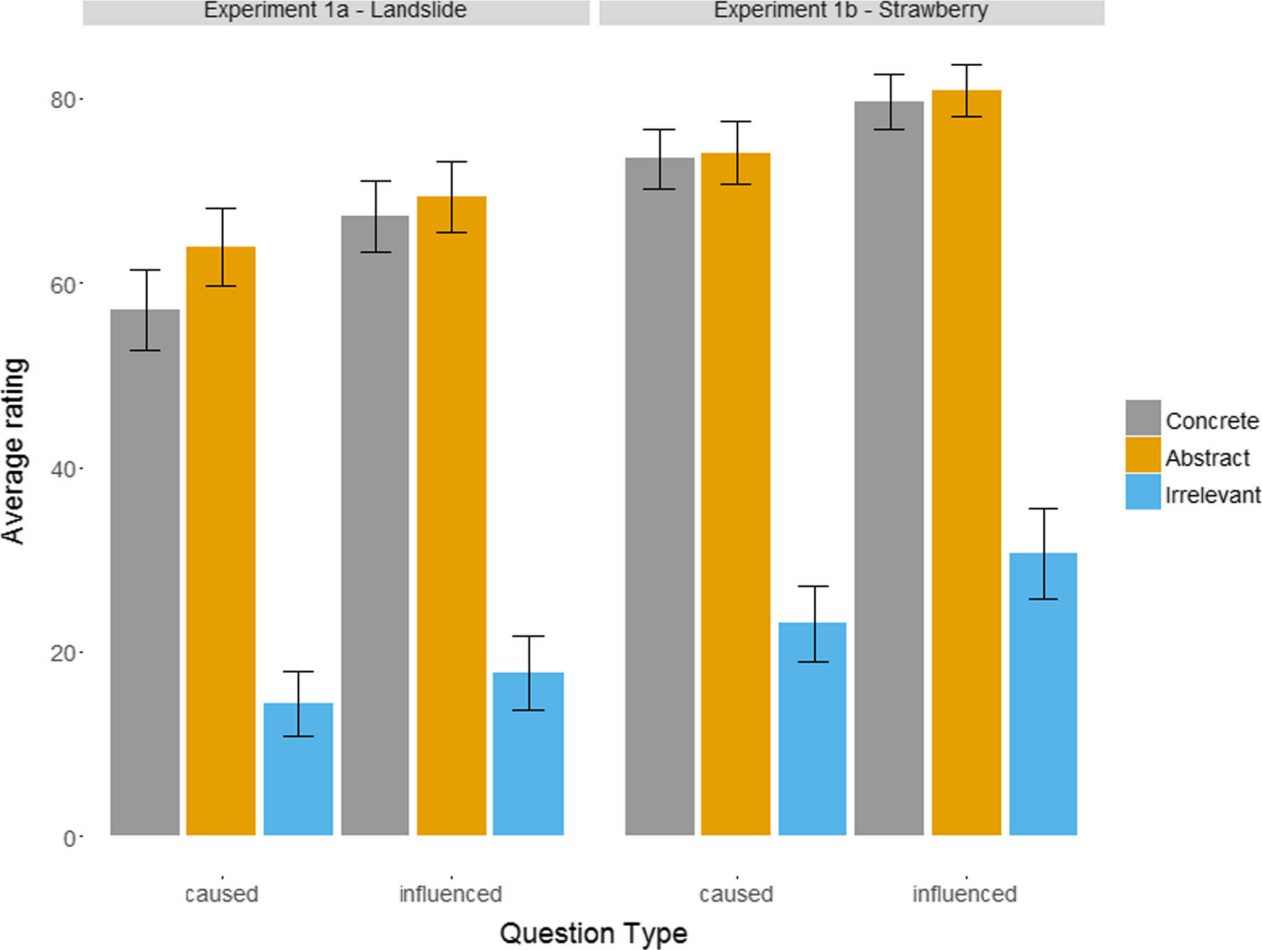
Mean values for causal ratings (i.e., “*X* caused the landslide/poor strawberry yield”) and causal influence ratings (i.e., “*X* affected the particular way in which the landslide happened”/“***X*** affected particular aspects of this year’s poor strawberry production”) averaged over participants

In both experiments, the same pattern of causal ratings and causal influence ratings was observed (see Fig. 3). Repeated-measures tests were highly significant on every occasion: *F*(1.7, 102.1) = 160.5, *p* < .001, η^2^ = .55, for causal ratings in Experiment 1a; *F*(1.62, 97.06) = 196.7, *p* < .001, for causal influence ratings in Experiment 1a; *F*(1.19, 71.15) = 189.8, *p* < .001, η^2^ = .62, for causal ratings in Experiment 1b; and *F*(1.21, 72.65) = 141.8, *p* < .001, η^2^ = .6, for causal influence ratings in Experiment 1b.

Participants rated irrelevant factors significantly lower than concrete factors both when asked whether the factor caused the explanandum (*p* < .001 in both experiments) and when asked whether it influenced aspects of the explanandum (*p* < .001 in both experiments). Irrelevant factors were also rated lower than abstract factors in both direct causal questions (*p* < .001 in both experiments) and causal influence questions (*p* < .001 in both experiments).

Finally, although abstract factors were rated higher than concrete factors, this difference was generally not significant, with one exception: In Experiment 1a, when asked whether each of the factors caused the landslide, participants were more likely to report that abstract factors (fine particles, steep slope, sparse vegetation) were more causally responsible than concrete factors (particles with diameter 2/64 of an inch, 37 degrees slope, 13% vegetation coverage) at a significant level (*p* = .004).

### Discussion

In contrast to what difference-making accounts of explanation propose regarding abstraction (Hitchcock & Woodward, 2003; Strevens, 2007), the findings of Experiment 1 indicate a preference for detail, even in cases in which detail is not judged to be causally relevant to the explanandum.^4^ Explanations in which the causal terms were abstracted received consistently lower ratings than explanations mentioning exact values. Yet when asked about the causal role of the factors that featured in each explanation, participants rated concrete and abstract factors equally highly, with abstract factors receiving significantly higher causal ratings on one occasion. Similarly, participants did not penalize the presence of irrelevant factors in explanations, despite the fact that they judged those factors to have minimal causal relation to the explanandum.

One question arising from these results is the role of causation in the way people evaluate explanations. Given the long philosophical tradition connecting causation and explanation (Psillos, 2002; Salmon, 1984; Woodward, 2003), it is surprising that the potency of each factor to bring about the explanandum was not the main determinant of the way the explanations were evaluated. The abstract terms, although equally efficacious, resulted in weaker explanations, while, conversely, causally irrelevant factors did not weaken the explanations.

It might be argued that, at least for some comparisons, it was not concreteness per se that guided participants but the presence of numerical values as indication of explanation quality. For that reason, we conducted an additional experiment, not reported here, using the same materials as in Experiment 1 but where abstract explanations included numerical ranges. Thus, the term “low temperature,” for example, was changed to “low temperature (0–5 degrees Celsius).” Ratings for abstract explanations were unchanged, as were the overall comparisons that we have reported here.

There are several other ways to understand our findings. Concreteness might signal the expertise or the truthfulness of the person providing the explanation (Bell & Loftus, 1985, 1989). Alternatively, it might be that the description of the exact conditions in which an event occurred facilitates understanding other aspects of the situation, thus achieving better unification (Kitcher, 1981). For example, the fact that the accident one is trying to explain took place close to the hospital, although not causally relevant to the accident itself, might explain other aspects of the event, such as the swift arrival of the ambulance. Before further discussing ways to account for our results, the next set of experiments will reevaluate the current findings in a simpler and more controlled context.

## Experiments 2a and 2b

The second experiment aimed for a more controlled investigation of the surprising findings obtained in Experiment 1. That experiment had the benefit of ecological validity but had only two scenarios and used only explanations of natural phenomena. Finally, the explanations incorporated multiple factors that could have interacted in complex ways. With that in mind, the following experiments used short explanations for multiple everyday events that varied only a single factor and included social as well as physical phenomena.

### Participants and materials

Sixty-one participants were recruited through Amazon’s Mechanical Turk for Experiment 2a and 60 for Experiment 2b. The mean age was 33.0 years (*SD* = 9.2) in Experiment 2a and 34.6 years (*SD* = 11.2) in Experiment 2b. There were 25 females (41.0%) in Experiment 2a and 26 (43.3%) in Experiment 2b.

### Design and procedure

Experiment 2a compared concrete to irrelevant explanations, and Experiment 2b compared concrete to abstract explanations. In each case, the two explanations differed by a single detail.

Both experiments had a 12 (scenario) × 2 (explanation type) repeated-measures design. After a few introductory screens, participants were asked to rate two explanations for each of 12 everyday events.^5^ Each screen presented the description of the event to be explained, a question specifying the explanandum and two explanations, each followed by a slider ranging from *poor* to *excellent*. The order of the events to be explained was randomized as well as the left-right position of the two explanations for each event.

In Experiment 2a, the two explanation types (concrete and irrelevant) were identical apart from the fact that the irrelevant explanation contained an extra detail that had no causal connection to the explanandum. For example, one of the stories described Michael’s road accident, mentioning that Michael had drunk eight vodka shots and three glasses of gin and tonic at Joe’s bar and asking, “Why did Michael have an accident?” The concrete version explained the accident by saying that the eight vodka shots and the three glasses of gin and tonic that Michael consumed severely reduced his concentration and increased his reaction time. The irrelevant explanation was identical, except that it also mentioned that Michael consumed the drinks at Joe’s bar.

Experiment 2b compared concrete and abstract explanations for the 12 scenarios. While the concrete versions referred to the exact values or quantities that were mentioned in the scenarios, the abstract explanations used a higher level of description for the critical term. For example, in the story of Michael’s accident, the concrete explanation was identical to the one used in Experiment 2a, while the abstract explanation mentioned that Michael had consumed “an excessive amount of alcohol” instead of the particular drink types and quantities.

### Results

In Experiment 2a, the average rating for concrete explanations (*M* = 76.23, *SE* = .75) was higher than the rating for irrelevant explanations (*M* = 69.65, *SE* = .85). A repeated-measures ANOVA revealed a significant main effect for both the scenario, *F*(7.16, 429.8) = 3.94, *p* < .001, η^2^ = .02, and the explanation type, *F*(1, 60) = 9.61, *p* < .001, η^2^ = .02, as well as their interaction, *F*(7.99, 479.4) = 3.94, *p* < .001, η^2^ = .02. Although there were variations between scenarios, participants rated only two of 12 irrelevant explanations higher than their concrete counterparts.

Similarly, clear results were observed in Experiment 2b with participants rating concrete explanations (*M* = 77.07, *SE* = .85) higher than abstract explanations (*M* = 67.26, *SE* = .90). Again, a repeated-measures ANOVA revealed a significant main effect for both the scenario, *F*(8.40, 495.3) = 6.53, *p* < .001, η^2^ = .02, and the explanation type, *F*(1, 59) = 22.09, *p* < .001, η^2^ = .04, as well as their interaction, *F*(6.46, 381.2) = 6.22, *p* < .001, η^2^ = .04. The preference for concrete explanations was true for all but two scenarios.

### Discussion

The current results replicated people’s preference for concrete explanations but also showed that causally irrelevant details made explanations less appealing. In the vast majority of the scenarios, participants gave higher ratings to explanations that contained particular details rather than abstracted versions of those details. Unlike Experiment 1, explanations were rated significantly lower when they contained causally irrelevant information.

Apart from using different scenarios, there are two candidate explanations for the discrepancy between Experiments 1a and 2a in the way irrelevant details were treated. An important difference is that each irrelevant explanation in Experiments 1a and 1b contained three causally irrelevant details rather than one, as in Experiment 2a. Perhaps discarding three details removes too much information from the explanations, even if that information is not causally connected to the explanandum.

Alternatively, the simultaneous presentation of three (Experiment 1) rather than two (Experiment 2) competing explanations might have led to attraction effects (Huber, Payne, & Puto, 1982) in Experiment 1. A strong preference for concrete over abstract explanations in Experiment 1 might have increased the ratings for irrelevant explanations that contained the same concrete descriptions. This is consistent with the fact that the difference between concrete and irrelevant explanations in Experiment 2a was smaller than the difference between concrete and abstract explanations in Experiment 2b. Removing detail through abstraction may be less desirable than removing irrelevant detail.

Both proposed accounts, together with the fact that irrelevant explanations still received high ratings (significantly higher than the midpoint) and the reluctance to abstract away, suggest that concreteness and accuracy are valued properties of explanations. However, the possibility remains that good explanations must appeal to causality. After all, the critical causal property was presumably inferred even in the concrete explanations of Experiment 2. For example, “eight vodka shots and the three glasses of gin and tonic” is for most people an “excessive amount of alcohol.” So the former phrase both communicates the difference maker and provides specific details. But the expression of detail appears to be important too.

Admittedly, there are many ways to transform a concrete explanation to an abstract one, as there are a wide variety of details one can add to an abstract explanation. Our results are surely influenced by some of our choices. There is no easy way to counteract this issue besides conducting further work and testing more variables. In this direction, the next pair of experiments radically changes the type of concrete information that is included. We assess the role of causation by testing whether people continue to prefer concreteness even when critical causal properties are not communicated.

## Experiments 3a and 3b

The aim of the final set of experiments is twofold. First, we wish to see if people’s attitude to causally irrelevant details generalizes to a different set of scenarios. Second, we will assess people’s preference for concreteness over abstraction in cases where concrete details fail to transmit the causally critical properties, allowing us to evaluate the perceived importance of causality in explanation.

### Participants and material

There were 44 participants in Experiment 3a and 36 in Experiment 3b, recruited through Amazon’s Mechanical Turk. The mean age was 30.6 years (*SD* = 8.37) in Experiment 3a and 33.0 years (*SD* = 9.44) in Experiment 3b. There were 18 females (40.9%) in Experiment 3a and 10 (27.8%) in Experiment 3b.

### Design and procedure

Experiments 3a and 3b used a different set of scenarios,^6^ but the design was identical to Experiments 2a and 2b. However, in Experiment 3b, abstract and concrete explanations differed along a second dimension: In the absence of specialized knowledge, the concrete term did not communicate the causal properties that bring about the explanandum either because the term itself was obscure or because it used a relatively unknown scale. In contrast, the abstract explanation mentioned either the category or a qualitative property. For example, to explain a fire in a warehouse, the concrete explanation attributed the fire to the presence of *ethyl chloride* while the abstract explanation referred to a *highly flammable* material. Similarly, to explain someone’s respiratory problems, the concrete explanation mentioned the presence of carbon dioxide at *a level of 3,000 ppm* while the abstract explanation referred to a *very high* level of carbon dioxide.

### Results

In Experiment 3a, the concrete (*M* = 74.66, *SE* = .93) and irrelevant (*M* = 73.71, *SE* = 1.04) explanations received approximately equal ratings across scenarios. A repeated-measures ANOVA was significant only for scenario, *F*(7.42, 319.04) = 2.38, *p* = .02, η^2^=.01, and the interaction term, *F*(6.15, 264.7) = 5.23, *p* < .001, η^2^ = .04 but not for explanation type (*p* = .747). A closer look at individual scenarios shows that concrete explanations were rated higher in six of 12 cases.

In Experiment 3b, participants preferred abstract (*M* = 82.50, *SE* = 1.12) over concrete explanations (*M* = 68.18, *SE* = 1.33). There was a significant effect for the scenario, *F*(7.26, 254.2) = 3.93, *p* < .001, η^2^ = 0.02, the explanation type, *F*(1, 35) = 10.18, *p* = .003, η^2^ = 0.08, and their interaction, *F*(6.35, 222.4) = 4.52, *p* < .001, η^2^ = .04. Abstract explanations were preferred in 11 of 12 scenarios.

### Discussion

In Experiment 3a, irrelevant details were not penalized as was the case in Experiments 1a and 1b. This rules out accounts based on attraction effects or the number of details included in explanations, discussed earlier.

Experiment 3b shows that the preference for concreteness that was observed in previous experiments does not persist when the concrete terms fail to communicate the causal properties of the event that brought about the explanandum. Although concrete explanations are still rated significantly higher than the midpoint, people prefer explanations that convey causal information as predicted by causal accounts of explanation (Salmon, 1984; Woodward, 2003).

## General discussion

We have investigated the extent to which people prefer explanations that present an accurate account of the state of affairs at the time of the explanandum or, alternatively, whether abstraction improves the perceived quality of explanations. In our experiments, abstract explanations either removed information that had no causal relation to the explanandum or replaced precise terms with more abstract ones that highlighted difference-making properties.

In violation of certain philosophical prescriptions (Garfinkel, 1981; Hitchcock & Woodward, 2003; Strevens, 2007; Weslake, 2010), abstraction is not in itself a desirable feature of an explanation. It becomes the preferred option only when it improves the communication of causal properties. In our experiments, people show a tendency for concreteness as long as the causal properties are not obscured by technical terms. Therefore, although causality appears to be a necessary property of a good explanation, causal terms are not selected based on their difference-making properties but rather on how accurately they match the events that took place.

Because people attend to whether the explanation offers a causal property, one might expect that details that are not causally related to the explanandum would reduce an explanation’s judged quality. Our results on this question were mixed, but some conclusions can be drawn. In two out of three experiments presented here, participants rated irrelevant explanations as high as concrete ones. Even though in Experiment 2a explanations without irrelevant details were preferred, ratings for irrelevant explanations significantly exceeded the midpoint in every scenario that we have tried. Causally irrelevant information is not penalized as strongly as one might expect. Since recent philosophical prescriptions (Strevens, 2007) suggest the removal even of factors that exert causal influence on the explanandum, provided these are not difference makers, the reluctance of our participants to penalize even causally irrelevant factors indicates a misalignment between the proposed normative principles and actual everyday practice.

Our findings extend the endorsement of concreteness and detail beyond the domain of neuroscience (Weisberg et al., 2008). They also extend it beyond the scope of reductionism (Hopkins et al., 2016; Trout, 2008). The competing explanations in our experiments did not differ in level of analysis. For example, causally irrelevant details, such as the edibility of the vegetation at a hill where a landslide occurred (Experiment 1a), are not more microscopic but simply more descriptive of the events that are being explained, yet participants did not penalize their presence in explanations.

In contrast to the current set of studies, the majority of philosophical work is concerned with explanations of law-like regularities.^7^ As a result, rather than evaluating explanations for token events, like a landslide at a particular location, most normative accounts assess explanations of type events, such as the natural phenomenon of landslides. It remains an open question how people would evaluate explanations of such regularities and whether explanations that appealed only to difference makers would, in those cases, be preferred.

More generally, although the current results point toward a preference for detail, it is reasonable to assume that this might depend on a variety of factors, such as the type of phenomena and the type of concrete details, the way the explanation is abstracted or concretized, as well as the interests of the receiver and their assumed background knowledge. We already saw in Experiment 3b, for example, that concrete details are not preferred when they fail to communicate the critical causal properties. Similarly, it is plausible that for certain types of properties (e.g., functional properties) there could be a stronger preference for abstraction. All these are open possibilities with further work required to decide whether the bias for concreteness is in fact universal or whether a mixed model where detail is sometimes preferred and other times penalized is more appropriate.

Given our observations, a pressing question is what underlies the observed preference for concreteness that leads to deviations from philosophical prescriptions. Our experiments inspire a few conjectures. Abstract explanations are more generalizable by retaining only the essence of the causal mechanism (Jorland, 1994; Nowak, 1992) and facilitating prediction. Detailed explanations, on the other hand, provide more information about the particular instance. This is more useful for understanding and perhaps explaining additional aspects of the particular instance (Kitcher, 1981; Strevens, 2007). Therefore, it might be the case that preferences regarding explanations depend on one’s aims. For example, referring back to our car accident example, a policy maker aims to prevent further accidents, while an insurance agent aims to understand as many aspects of the accident as possible. The former might show a preference for abstract, generalizable explanations due to their potentially preventative role (Garfinkel, 1981), while the latter might prefer explanations with concrete details that can prove useful when processing the insurance claim. Thus, the tendency toward precision might be explained by participants defaulting to a more backwards-looking stance toward explanations.

Similarly, irrelevant details might have helped participants visualize how the explanandum came to be. The fact, for example, that someone suffered from respiratory problems is adequately explained by his exposure to high levels of carbon dioxide, irrespective of whether or not this exposure took place in the school where he works (Experiment 3a). This causally irrelevant information, however, helps one imagine the mechanism that led to the explanandum, which by itself might promote a feeling of understanding and, moreover, explain why children were endangered or why a legal case was brought against the builder.

What is apparent is that people do not adhere to the normative principles put forward by some philosophers. Explanations in everyday usage serve goals beyond uncovering how an explanandum came to be. These goals are better served through detail and accuracy, making abstraction a less than ideal option.

## Appendix A: Stimuli used in Experiments 2a and 2b

1. Exam
  Peter failed his bar examination. He used to study on average for 30 minutes per day in his office.
  - Why did Peter fail the exam?
    - Concrete: Because studying for 30 minutes per day on average was insufficient for understanding and remembering the various topics on which he was examined.
    - Abstract: Because studying for less than 5 hours a day on average was insufficient for understanding and remembering the various topics on which he was examined.
    - Irrelevant: Because studying for 30 minutes per day on average in his office was insufficient for understanding and remembering the various topics on which he was examined.
2. Laptop
  Mary’s laptop failed to start after she spilled a bottle containing 340 ml of sparkling water on the keyboard.
  - Why did Mary’s laptop fail to start?
    - Concrete: Because the 340 ml of water that was spilled on the keyboard reached the laptop’s battery and reacted with the battery’s alkaline elements, thus destroying the laptop.
    - Abstract: Because the large amount of water that was spilled on the keyboard reached the laptop’s battery and reacted with the battery’s alkaline elements, thus destroying the laptop.
    - Irrelevant: Because the 340 ml of sparkling water that was spilled on the keyboard reached the laptop’s battery and reacted with the battery’s alkaline elements, thus destroying the laptop.
3. Holidays
  Paul did not enjoy his holidays. During his stay there were winds with average speed of 46 mph around his 5-star resort.
  - Why did not Paul enjoy his holidays?
    - Concrete: Because the 46-mph winds blowing around his resort prevented him from swimming in the sea or, even, relaxing at the beach.
    - Abstract: Because the strong winds blowing around his resort prevented him from swimming in the sea or, even, relaxing at the beach.
    - Irrelevant: Because the 46-mph winds blowing around his 5-star resort prevented him from swimming in the sea or, even, relaxing at beach.
4. Plant
  John’s plant died. The plant that was decorating John’s bedroom was left without water for 300 days.
  - Why did John’s plant die?
    - Concrete: Because plants need water to photosynthesize (i.e., to create their food), and John’s plant was left without water for 300 days; thus, it starved.
    - Abstract: Because plants need water to photosynthesize (i.e., to create their food), and John’s plant was left without water for more than 60 days; thus, it starved.
    - Irrelevant: Because plants need water to photosynthesize (i.e., to create their food), and John’s plant, which was decorating his bedroom, was left without water for 300 days; thus, it starved.
5. Cake
  Lucia’s cake was a disaster. The cake has been in Lucia’s oven for 9 hours.
  - Why was Lucia’s cake ruined?
    - Concrete: Because the cake was left in the oven for 9 hours, and thus it was completely burnt.
    - Abstract: Because the cake was left in the oven for a very long time, and thus it was completely burnt.
    - Irrelevant: Because the cake was left in Lucia’s oven for 9 hours, and thus it was completely burnt.
6. Accident
  Michael had a road accident. He had drunk eight vodka shots and three glasses of gin and tonic at Joe’s bar.
  - Why did Michael have an accident?
    - Concrete: Because the eight vodka shots and the three glasses of gin and tonic that Michael consumed severely reduced his concentration and increased his reaction time.
    - Abstract: Because the excessive amount of alcohol that Michael consumed severely reduced his concentration and increased his reaction time.
    - Irrelevant: Because the eight vodka shots and the three glasses of gin and tonic that Michael consumed at Joe’s bar severely reduced his concentration and increased his reaction time.
7. Job
  Kevin’s application for a job was unsuccessful. He sent his application via priority mail 35 days after the application deadline.
  - Why was Kevin’s application unsuccessful?
    - Concrete: Because his application was sent 35 days after the deadline, and thus he was not even considered for the position.
    - Abstract: Because his application was sent long after the deadline, and thus he was not even considered for the position.
    - Irrelevant: Because his application was sent via priority mail 35 days after the deadline, and thus he was not even considered for the position.
8. Lake
  Llyn Dinas, a lake near Beddgelert in north Wales, has frozen. The water temperature in the lake that is famous for its trout fishing was 14 degrees Fahrenheit.
  - Why did the lake freeze?
    - Concrete: Because, since the temperature of the lake was 14 degrees Fahrenheit, the lake’s water molecules were all locked together, forming ice.
    - Abstract: Because, since the temperature of the lake was below 32 degrees Fahrenheit, the lake’s water molecules were all locked together, forming ice.
    - Irrelevant: Because, since the temperature of the lake that is famous for its trout fishing was 14 degrees Fahrenheit, the lake’s water molecules were all locked together, forming ice.
9. Bleeding
  Tina’s finger was bleeding heavily. She had cut her finger ¼ inches deep with a kitchen knife.
  - Why did Tina’s finger bleed heavily?
    - Concrete: Because the ¼-inch deep cut damaged blood vessels that carry blood under high pressure.
    - Abstract: Because the nonsuperficial cut damaged blood vessels that carry blood under high pressure.
    - Irrelevant: Because the ¼-inch deep cut by the kitchen knife damaged blood vessels that carry blood under high pressure.
10. Inflation
  The rate of inflation has increased. Earlier in the year, the left-wing government has raised the sales tax by 10%.
  - Why did the rate of inflation increase?
    - Concrete: Because the 10% raise of the sales tax has led to higher prices of consumer goods, which is essentially what inflation measures.
    - Abstract: Because the significant raise of the sales tax has led to higher prices of consumer goods, which is essentially what inflation measures.
    - Irrelevant: Because the 10% raise of the sales tax by the left-wing government has led to higher prices of consumer goods, which is essentially what inflation measures.
11. Battle
  In the Battle of Thermopylae in 480 BC, the Greeks were defeated by the Persians. In the battle, that took place close to the city of Trachis, the Greek army was about 7,000 men while the Persian army numbered 130,000 soldiers.
  - Why was the Greek army defeated?
    - Concrete: Because during the battle, the Persian army outnumbered the Greek army by 123,000 soldiers.
    - Abstract: Because during the battle, the Persian army vastly outnumbered the Greek army.
    - Irrelevant: Because during the battle that took place close to the city of Trachis, the Persian army outnumbered the Greek army by 123,000 soldiers.
12. Elections
  In the last German elections, the Free Democrats (FDP) failed to meet the 5% vote threshold required to enter the parliament. The party, which was established in 1948, secured 4.8% of the vote.
  - Why did the FDP fail to enter the German parliament?
    - Concrete: Because the party won 0.2% fewer votes than required to exceed the threshold.
    - Abstract: Because the party won fewer votes than required to exceed the threshold.
    - Irrelevant: Because the party that was established in 1948 won 0.2% less than required to exceed the threshold.

## Appendix B: Stimuli used in Experiments 3a and 3b

1. Respiratory
  Peter was suffering from respiratory problems. The concentration of carbon dioxide in the school where he was teaching was regularly at the very high level of 3,000 ppm.
  - Why was Peter suffering from respiratory problems?
    - Concrete: Because he was regularly exposed to carbon dioxide at the level of 3,000 ppm.
    - Irrelevant: Because he was regularly exposed to carbon dioxide at the level of 3,000 ppm at the school where he was teaching.
    - Abstract: Because he was regularly exposed to a very high level of carbon dioxide.
2. Club
  Melissa had a headache after leaving the night club. The music in the recently renovated club was playing extremely loudly at 120 dBA SPL.
  - Why did Melissa have a headache?
    - Concrete: Because the music in the club was playing at 120 dBA SPL.
    - Irrelevant: Because the music in the recently renovated club was playing at 120 dBA SPL.
    - Abstract: Because the music in the club was playing extremely loudly.
3. Welfare
  Hospitals across the country struggled to cope with demand. The government had severely reduced the health-care budget by 2.4%, contrary to what was promised before the elections.
  - Why did the hospitals struggle to cope with demand?
    - Concrete: Because the government had reduced the health-care budget by 2.4%.
    - Irrelevant: Because, contrary to what was promised before the elections, the government had reduced the health-care budget by 2.4%.
    - Abstract: Because the government had severely reduced the health-care budget.
4. Holidays
  Paul did not enjoy his holidays. During his stay, there were strong winds with average speed of 46 mph blowing from the west, which prevented Paul from enjoying the beach.
  - Why did not Paul enjoy his holidays?
    - Concrete: Because the 46-mph winds prevented him from enjoying the beach.
    - Irrelevant: Because the 46-mph winds blowing from the west prevented him from enjoying the beach.
    - Abstract: Because the strong winds prevented him from enjoying the beach.
5. Flood
  Larry’s house was flooded. The Yare river, which is nearby, has overflown for the third time in that year.
  - Why was Larry’s house flooded?
    - Concrete: Because Yare, which is nearby, could not withhold the water.
    - Irrelevant: Because Yare, which is nearby, could not withhold the water for the third time in that year.
    - Abstract: Because a river, which is nearby, could not withhold the water.
6. Medical
  Daniel was stressed. The outcome of the scintigraphy medical test that he had 5 days earlier was positive.
  - Why was Daniel stressed?
    - Concrete: Because the outcome of his scintigraphy was positive.
    - Irrelevant: Because the outcome of the scintigraphy that he had 5 days earlier was positive.
    - Abstract: Because the outcome of his medical test was positive.
7. Nausea
  Mary was feeling very nauseated. She had accidentally ingested 8 grams of paracetamol, much more than the recommended maximum daily dose.
  - Why was Mary feeling very nauseated?
    - Concrete: Because she had ingested 8 grams of paracetamol.
    - Irrelevant: Because she had accidentally ingested 8 grams of paracetamol.
    - Abstract: Because she had overdosed on paracetamol.
8. Landslide
  There was a landslide at the hill close to David’s house. Throughout the cold winter day, intense rain was falling at the rate of 0.63 inches per hour.
  - Why was there a landslide close to David’s house?
    - Concrete: Because, throughout the day, rain was falling at the rate of 0.63 inches per hour.
    - Irrelevant: Because, throughout the cold winter day, rain was falling at the rate of 0.63 inches per hour.
    - Abstract: Because, throughout the day, intense rain was falling.
9. Fire
  A large fire destroyed the warehouse. Around noon on a very hot day, at a time when many people were still working, a large quantity of the very flammable ethyl chloride was being transported in the warehouse.
  - Why was there a fire in the warehouse?
    - Concrete: Because a large quantity of ethyl chloride was being transported in the warehouse at a time when the temperature was very high.
    - Irrelevant: Because a large quantity of ethyl chloride was being transported in the warehouse at a time when the temperature was very high and many people were still working.
    - Abstract: Because a large quantity of a very flammable material was being transported in the warehouse at a time when the temperature was very high.
10. Strawberry
  Barbara’s strawberry yield was very poor that year. The strawberries that were planted in early autumn attracted the leaf-eating tarsonemid mite.
  - Why was Barbara’s strawberry yield poor that year?
    - Concrete: Because her strawberries attracted the tarsonemid mite.
    - Irrelevant: Because her strawberries that were planted in early autumn attracted the tarsonemid mite.
    - Abstract: Because her strawberries attracted a leaf-eating mite.
11. TV
  Kevin’s TV had no picture even though it did have sound. The cold-cathode florescent lamp, a component present in LCD TVs and used to generate light, was malfunctioning.
  - Why did Kevin’s TV have no picture even though it did have sound?
    - Concrete: Because the cold-cathode florescent lamp was malfunctioning.
    - Irrelevant: Because the cold-cathode florescent lamp, a component present in LCD TVs, was malfunctioning.
    - Abstract: Because the component used to generate light was malfunctioning.
12. Accident
  John had a car accident. He was driving after having drunk three glasses of tsikoudia, a very strong alcoholic drink made from pomace.
  - Why did John have an accident?
    - Concrete: Because he was driving after having drunk three glasses of tsikoudia.
    - Abstract: Because he was driving after having drunk three glasses of a very strong alcoholic drink.
    - Irrelevant: Because he was driving after having drunk three glasses of tsikoudia made from pomace.

## Footnotes

1 By this definition, we are intentionally exaggerating what philosophers, especially those adopting a causal account of explanation, designate as explanatorily irrelevant information. Our “irrelevant” explanations include details with no plausible causal relation to the explanandum, in an attempt to generate more pronounced differences between the explanations that participants were presented.

2 In certain circumstances, people might prefer reductionist or concrete explanations because the explanandum warrants such a choice (i.e., because the explanatory work is in fact achieved by lower level or more concrete details). In this manuscript, however, we focus on information that most people would agree has no influence on the explanandum.

3 While designing the experiment we had to rely on our intuitions regarding what type of facts are irrelevant to the explanandum. Given that participants could very well disagree with these intuitions, we asked them to rate the causal relevance of each factor at the end of the experiment, as will be shortly discussed.

4 Some important differences between the types of explanations that commonly appear in philosophical discourse and the current work will be discussed in the General Discussion section.

5 The complete list of scenarios and explanations used in Experiments 2a and 2b can be found in Appendix A.

6 The complete list of scenarios and explanations used in Experiments 3a and 3b can be found in Appendix B.

7 Strevens, however, thinks the process of abstraction yields superior explanations not only of laws but also of “ongoing states of affairs, ‘effects’, and other generalizations” (Strevens, 2007, p. 7).
